# Kouprey (*Bos sauveli*) genomes unveil polytomic origin of wild Asian *Bos*

**DOI:** 10.1016/j.isci.2021.103226

**Published:** 2021-10-06

**Authors:** Mikkel-Holger S. Sinding, Marta M. Ciucani, Jazmín Ramos-Madrigal, Alberto Carmagnini, Jacob Agerbo Rasmussen, Shaohong Feng, Guangji Chen, Filipe G. Vieira, Valeria Mattiangeli, Rajinder K. Ganjoo, Greger Larson, Thomas Sicheritz-Pontén, Bent Petersen, Laurent Frantz, M. Thomas P. Gilbert, Daniel G. Bradley

**Affiliations:** 1 Smurfit Institute of Genetics, Trinity College Dublin, Dublin, Ireland; 2 Globe Institute, University of Copenhagen, Copenhagen, Denmark; 3 School of Biological and Chemical Sciences, Queen Mary University of London, London, UK; 4 Laboratory of Genomics and Molecular Medicine, Department of Biology, University of Copenhagen, Copenhagen, Denmark; 5 BGI-Shenzhen, Beishan Industrial Zone, Shenzhen, China; 6 University of Chinese Academy of Sciences, Beijing, China; 7 Department of Geology, University of Jammu, Jammu, India; 8 The Palaeogenomics and Bio-Archaeology Research Network, Research Laboratory for Archaeology and History of Art, University of Oxford, Oxford, UK; 9 Centre of Excellence for Omics-Driven Computational Biodiscovery (COMBio), Faculty of Applied Sciences, AIMST University, Kedah, Malaysia; 10 Palaeogenomics Group, Department of Veterinary Sciences, Ludwig Maximilian University, Munich, Germany; 11 Center for Evolutionary Hologenomics, University of Copenhagen, Copenhagen, Denmark; 12 Norwegian University of Science and Technology, University Museum, Trondheim, Norway

**Keywords:** Biological sciences, Evolutionary biology, Phylogenetics

## Abstract

The evolution of the genera *Bos* and *Bison*, and the nature of gene flow between wild and domestic species, is poorly understood, with genomic data of wild species being limited. We generated two genomes from the likely extinct kouprey (*Bos sauveli*) and analyzed them alongside other *Bos* and *Bison* genomes. We found that *B. sauveli* possessed genomic signatures characteristic of an independent species closely related to *Bos javanicus* and *Bos gaurus*. We found evidence for extensive incomplete lineage sorting across the three species, consistent with a polytomic diversification of the major ancestry in the group, potentially followed by secondary gene flow. Finally, we detected significant gene flow from an unsampled Asian *Bos*-like source into East Asian zebu cattle, demonstrating both that the full genomic diversity and evolutionary history of the *Bos* complex has yet to be elucidated and that museum specimens and ancient DNA are valuable resources to do so.

## Highlights

• We generated two genomes from the likely extinct kouprey (*Bos sauveli*)
• Extensive *mt* and nuclear-genome-wide incomplete lineage sorting across wild Asian *Bos*
• Initial polytomic diversification of the wild Asian *Bos*—kouprey, banteng, and gaur

## Graphical abstract

**Figure:**
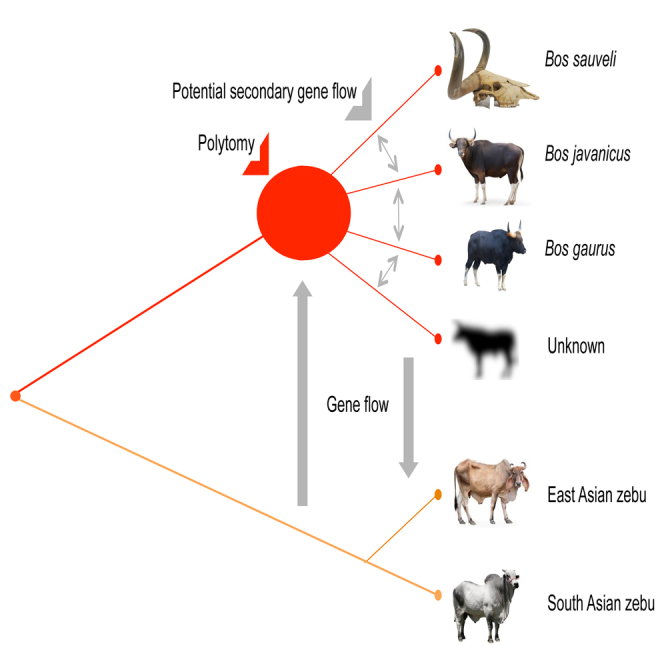

## Introduction

The genera *Bos* and *Bison* form a complex group of closely related wild species including several domesticated forms (Table 1). Previous studies of the group using whole nuclear genome sequences revealed clear differentiation between species and substantial admixture, especially in the domestic groups (Qiu et al., 2015; Medugorac et al., 2017; Chen et al., 2018; Wu et al., 2018). Asia is home to the largest diversity of wild *Bos* species, from which the critically endangered and likely extinct kouprey (Timmins et al., 2016), *Bos sauveli*, has never been investigated from a genome-wide perspective. Various conflicting hypotheses have previously been suggested regarding its taxonomy and phylogenetic placement, including that it represents a private lineage (Coolidge, 1940; Wharton, 1957), related to gaur *Bos gaurus* and/or banteng *Bos javanicus* (Urbain, 1937; Bohlken, 1961; Pfeffer and Kim-San, 1967; Geraads, 1992; Hassanin and Ropiquet, 2004), aurochs *Bos primigenius* (Groves, 1981), or domestic cattle (Bohlken, 1963). Alternatively, other hypotheses argue it may have originated as a hybrid, for example, between *B. javanicus* and zebu cattle (Edmond-Blanc, 1947; Bohlken, 1958; Galbreath et al., 2006) (but see Hassanin and Ropiquet, 2007a; Galbreath et al., 2007; Hedges et al., 2007), or between *B. javanicus* and either *B. gaurus* or water buffalo *Bubalus bubalis* (Edmond-Blanc, 1947).

**Table 1:** Overview of *Bos* and *Bison* genomes investigated in the study, as well as correlation of wild type to domestic forms

Wild species	# Wild samples	Domesticate	# Domestic samples
*Bos sauveli*	2	NA	NA
*Bos javanicus*	3	Bali cattle	3
*Bos gaurus*	3	Gayal	3
*Bos mutus*	3	Domestic yak	3
*Bison bonasus*	5	NA	NA
*Bison bison*	3	NA	NA
*Bos primigenius*	3	Taurine & zebu cattle	9 & 12

The first and second columns represent the species diversity and the number of samples for each species in the dataset. The third and fourth columns represent the domesticated forms (if present) for each wild species and the number of individuals in the dataset.

See also Table S1 for full nuclear genomes and additional mitochondrial data about the samples.

Although both its presence in the Pleistocene fossil record (Vithayanon and Bhumpakphan, 2004) and the results of a genetic analysis of eight loci (three autosomal, two Y chromosome, and three mitochondrial) (Hassanin and Ropiquet, 2007b) have been used to support the argument that *B. sauveli* is a distinct, non-hybrid lineage within the genus *Bos*, the ability of such analyses to reconstruct species trees and assess levels of potential hybridization is much less than can be achieved using nuclear genome-scale datasets.

**Figure 1:**
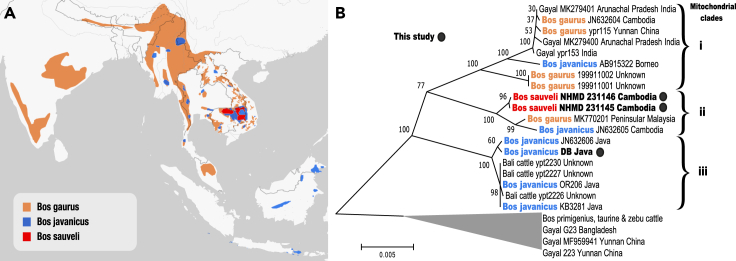
Distribution of wild Asian *Bos* and mitochondrial phylogeny (A) Current geographic range based on IUCN data, *B. sauveli* (possibly extinct)*, B. gaurus* (extant), and *B. javanicus* (extant, possibly extant and possibly extinct) (Duckworth et al., 2016; Gardner et al., 2016; Timmins et al., 2016). (B) Mitochondrial phylogeny rooted to the *B. primigenius*, taurine, and zebu cattle clade. Bootstrap support is given at the base of nodes. See also Figure S2 and Table S1.

Therefore, to close a lacuna in the *Bos* genome catalog and investigate the evolution of *B. sauveli*, we sequenced nuclear and mitochondrial-wide genome data of two specimens collected in Cambodia (Figure 1A) in 1957 and housed in the Natural History Museum of Denmark, and the genome of a *B. javanicus* specimen collected in 1991 from Whipsnade Zoo in the United Kingdom (MacHugh, 1996).

## Results and discussion

### Mitochondrial paraphyly

We performed shotgun sequencing on sub-samples from skulls (Figure S1) of the two *B. sauveli* (NHMD 231145-6) and hair shafts of the *B. javanicus* (DB). The *B. sauveli* material was processed in an ancient DNA facility following standard practice guidelines (Orlando et al., 2013). We sequenced *ca* 970M reads for the sample NHMD 231145, 1.610M reads for NHMD 231146, and 145M reads for the sample DB (Table S1, mapping stats). Sequence data (see key resources table, deposited data) were mapped to the water buffalo (*B. bubalis*) reference genome UMD_CASPUR_WB_2.0 (Williams et al., 2017) We used this outgroup as reference, to avoid potential mapping biases in downstream ancestry analyses (Gopalakrishnan et al., 2017). The nuclear genomes of the two *B. sauveli* samples had a coverage of 1.8x (NHMD 231145) and 3.6x (NHMD 231146), whereas the *B. javanicus* had 1.5x coverage.

Mitochondrial genome assemblies were obtained by mapping the raw sequencing data to both the nuclear and mtDNA sequences of the taurine cattle reference genome bostau9 (Rosen et al., 2020). We aligned the shotgun reads to the full genome to remove potential numts (nuclear mitochondrial DNAs) from the assembly (Castruita et al., 2015). The mitochondrial genomes of the two *B. sauveli* samples had a coverage of 126.75x (NHMD 231145) and 163.99x (NHMD 231146), whereas the *B. javanicus* (DB) was recovered at 50.10x coverage. To place our findings in the context of other published studies, we first investigated the mitochondrial data, by combining our newly generated sequences with other data from 42 *Bos* specimens (Table S1) that include near-complete mitochondrial genomes as well as *cytochrome b* fragments.

Modeled in a series of neighbor-joining (NJ) trees (Figures 1B and S2), our newly generated *B. sauveli* sequences cluster together with a previously published *cytochrome b* sequence generated from the *B. sauveli* holotype (Hassanin and Ropiquet, 2004) (*Muséum National d'Histoire Naturelle*), corroborating the previous conclusion that the *B. sauveli* possesses a distinct mitochondrial lineage (Hassanin and Ropiquet, 2007a). In the wider context, the trees place *B. sauveli*, *B. javanicus*, and *B. gaurus* in three major clades: (1) *B. gaurus*, gayal, and Bornean *B. javanicus,* (2) a *B. sauveli* clade sister to a clade of Cambodian *B. javanicus* and a *B. gaurus* from Peninsular Malaysia, (3) Javan *B. javanicus* and Bali cattle.

Although this overall structure reflects the results of previous analyses that used smaller individual datasets (Hassanin and Ropiquet, 2007a, 2007b; Saijuntha et al., 2013; Ishige et al., 2016; Rosli et al., 2019), our near-complete mitochondrial genome approach highlights a pronounced paraphyly of *B. javanicus* and *B. gaurus.* There is, however, clear geographic structure and private species lineages, suggesting that this lack of monophyly is the result of ancient (rather than recent) hybridization and/or incomplete mitochondrial lineage sorting. In addition, our analyses also enabled us to obtain insights into the origin of captive and domestic specimens. The mitochondrial phylogenies support zoo records of a Javan origin of the reference *B. javanicus* (see STAR Methods, sample description). But interestingly, the two *B. gaurus* from Omaha's Henry Doorly Zoo (ID 199911001-2) possess a private divergent haplotype, basal in clade (2) (Figure 1B) to *B. gaurus* and gayal from East India and Southeast Asia, as well as Bornean *B. javanicus*. The geographic origin of these two specimens is unknown (see STAR Methods, sample description), but based on Cytb diversity, they may represent Southwest Indian *B. gaurus* diversity (Figure S2). Finally, several gayal sequences fall among sequences of zebu cattle (Figures 1B and S2), likely as a result of recent introgression from cattle.

### Species structure with genome-wide incomplete lineage sorting

Next, we took advantage of a comprehensive species level dataset of *Bos* nuclear genomes to investigate the initial structure and relatedness of the species. We first computed principal components analysis (PCA; Figure S3), including wild-type and domesticated forms. Orientated from the origin of the plot (the cross 0.0 of PC 1 and 2), the variation distributes individuals along three primary trajectories: (1) cattle and *B. primigenius*; (2) *Bos bison, Bos bonasus, Bos mutus*, and domestic yak; (3) *B. sauveli, B. gaurus*, gayals, plus *B. javanicus* and Bali cattle (Asian *Bos*). The very tight clustering of both *B. sauveli* samples suggests that they are a homogeneous group, placed within the trajectory with *B. gaurus* and *B. javanicus*.

Of interest, East and South Asian zebu differentiate from each other, with East Asian zebu placed toward Asian *Bos*. Previous studies have concluded that East Asian zebu possess significant introgressed genomic diversity from an incompletely described exotic source (Wangkumhang et al., 2015; Chen et al., 2018), and our PCA analysis confirms that zebu do not form an homogeneous group. Bali cattle and domestic yak fall close to their wild-type counterparts, *B. javanicus* and *B. mutus*, respectively. However, the gayal displayed more heterogeneity, with one falling with *B. gaurus*, whereas two other specimens were closer to cattle and *B. primigenius*, likely driven by cattle admixture in these two individuals and corroborating mitochondrial results.

**Figure 2:**
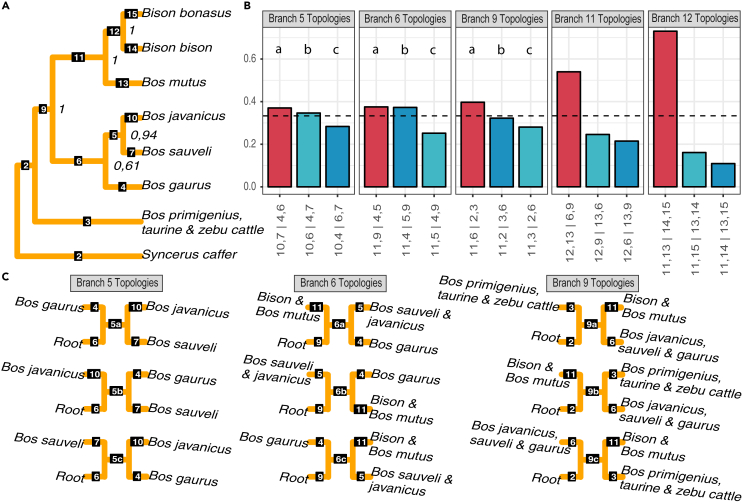
Species tree and incomplete lineage sorting (A) Nuclear genome phylogeny estimated by ASTRAL-III, with monophyletic clusters collapsed into a single leaf node. The tree is rooted to the African buffalo (*Syncerus caffer)* and includes only wild-type genomes, with the exception of cattle, see also Figure S1 and Table S1. Numbers placed at branch nodes represent clade supports expressed in posterior probabilities and computed by RAxML-ng using 100 replicates in Astral-III. (B) Display of quartet frequencies of the three possible configurations of internal branches in the nuclear phylogeny, when evaluating clades as an underlying unrooted tree. Red bars show the topscore configuration presented in the phylogeny (A), whereas the two blue bars show alternative configurations. Alternative tree configurations are labeled corresponding to branch IDs in (A). The dotted line indicates a level of a one-third bipartition for every quartet, which is the threshold frequency of a true bipartition (Allman et al., 2011). (C) Visualization of alternative topologies for branches 5, 6, and 9 in (A) and (B).

To interrogate the confidence of the phylogenetic position of *B. sauveli*, we generated an ASTRAL-III phylogeny (Zhang et al., 2018). Previous genetic research has demonstrated that there is cattle introgression into domestic yak, Bali cattle, and gayals (Wu et al., 2018), which potentially could induce noise in the analysis. Therefore, with the exception of domestic cattle, we restricted this analysis to the undomesticated *Bos* forms. The phylogeny (Figures 2A and S3) is based on 1,000 individual trees, each built on 1,000 randomly selected sequences of 5,000 bp each and 100 bootstrap replicates computed using RAxML (Stamatakis, 2014). All species-level clades were supported by a maximum posterior probability score of 1.00 except for *B. bonasus*, which instead possessed a posterior probability of 0.90 (Figure S3). The evolution and cladistics of *B. bonasus* is known to be complex and may be influenced by gene flow from cattle, or from a lineage ancestral to cattle and/or by incomplete lineage sorting (Gower et al., 2016; Węcek et al., 2016; Grange et al., 2018; Wang et al., 2018).

Overall, this phylogeny mirrors the structure of the PCA and supports previous genome-based trees (Chen et al., 2018; Wu et al., 2018), with the addition of *B. sauveli* falling as a sister clade to *B. javanicus* in a clade adjacent to *B. gaurus*. The posterior probability support of the *B. sauveli-B. javanicus* clade was 0.94, and the support of the whole Asian *Bos* clade, when including *B. gaurus*, was only 0.61 (Figure 2A). To evaluate incomplete lineage sorting and the support for alternative topologies, we re-used the ASTRAL data of 1,000 individual 5,000-bp sequences from each specimen in a DiscoVista analysis (Sayyari et al., 2018) (Figures 2B and 2C).

The monophyly of *B. bison* and *B. bonasus* is well supported. However, when compared with *B. bison*, a slightly higher ratio of sequences place *B. bonasus* outside the *bison*-*B. mutus* clade, supporting partial discrepancy in the evolution of *B. bison* and *B. bonasus*. There is further strong support for the placement of *B. mutus* as sister to *Bison*, with a slightly higher affinity to the clade of the Asian *Bos*, compared with the *Bison* genomes. Cattle and *B. primigenius* are confidently placed as the outgroup to the other *Bos* and *Bison*, however, with slightly higher affinity to the clade of Asian *Bos*, compared with the *Bison-B. mutus* clade. Of note, the internal branch (branch 9, Figure 2A) separating cattle and the root from the other *Bos* and *Bison* is short, potentially reflecting incomplete lineage sorting and complex evolutionary relationships across these species.

The posterior probability of 0.61 supporting the individual ASTRAL tree placing *B. gaurus* as sister to the *B. sauveli-B. javanicus* clade is reflected in the DiscoVista results, showing a near-equal proportion of trees placing *B. gaurus* in a clade with the *bison-B. mutus* as with the *B. sauveli-B. javanicus*. The most frequent topology places *B. sauveli* as sister clade of *B. javanicus*. However, a similar proportion of sequences support the topology where *B. gaurus* and *B. sauveli* form a clade, with the configuration placing *B. javanicus* and *B. gaurus* as a clade being the least frequent, but still supported by nearly a third of the sequences. The ASTRAL-DiscoVista combination clearly highlights that the complex phylogenetic relationships of wild Asian *Bos* cannot be modeled using bifurcating trees but rather support a polytomic relationship of the major ancestry in *B. javanicus*, *B. gaurus*, and *B. sauveli*, a diversification that would corroborate the paraphyly observed in mitochondrial lineages.

### Polytomy in deep ancestry of wild Asian *Bos* and ghost admixture into East Asian zebu

**Figure 3:**
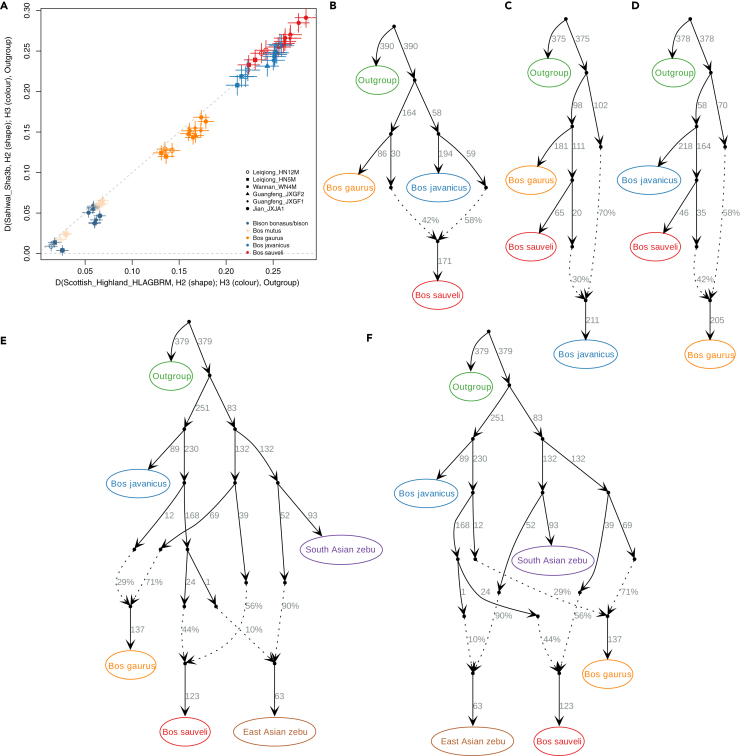
Ghost admixture into East Asian zebu (A) Pairwise D-statistics testing allele sharing between East Asian zebu and out groups, compared with South Asian zebu and taurine cattle. Increases along x and/or y axes indicate H2-H3 allele sharing. Horizontal and vertical bars indicate 3 standard errors for the D-statistic tests in the x and y axes, respectively. (B–F) qpBrute admixture graph based on all pairwise D statistics between included samples, using *S. caffer* as root. Solid lines indicate genetic drift; dotted lines indicate gene flow. (B–D) The three species involved show lack of clear structure and may be products of ancient hybrid origins. (E and F) Two examples of 10 (additional 8 in Figure S4) qpBrute admixture graphs fitting the specific dataset. Tests are expanding on the models of (B–D), with inclusion of zebus. East Asian zebu attract divergent gene flow, not directly explained by any included wild species.

Finally, we focused on the East Asian zebu to explain their marked divergence from other cattle. Zebu cattle descend from *B. primigenius* that were indigenous to modern India and Pakistan (Chen et al., 2010). South Asian zebu likely retain this original ancestry, whereas East Asian zebu likely diverged after introduction to their locale around ∼2,500 years ago (Higham, 1996; Chen et al., 2010), involving introgression from an incompletely described exotic source (Chen et al., 2018). The ASTRAL phylogeny finds that East Asian zebu fall in individual branches basal to two monophyletic sister clades of, respectively, (i) South Asian zebu and (ii) taurine cattle and West Eurasian *B. primigenius* (Figure S3). However, East Asian zebu clusters tightly in PCA (Figure S3), suggesting that they have diverse ancestral components but from similar ancestral sources, outside the diversity of the reference South Asian zebu, taurine cattle, and *B. primigenius*. Using *D-*statistics, we tested for excessive allele sharing between East Asian zebu and other *Bos* and *Bison* diversity when compared with other cattle. We found that all East Asian zebu in the dataset have significantly higher allele sharing with *B. sauveli*, closely followed by *B. javanicus* (Figures 3A and S4), a pattern that could be driven by introgression from both.

To investigate the more complex admixture patterns among and between zebu and Asian *Bos*, we used *qpBrute* (Liu et al., 2019) to model admixture graphs of the genomes. Initially, we tested relationships among *B. sauveli*, *B. javanicus*, and *B. gaurus* (run ID Wild_1 Table S2); surprisingly, the data allow three equally best supported ancestral scenarios, where each species alternately is the product of a hybridization event (Figures 3B–3D). Although this illustrates that the genealogy is elusive, it corroborates the incomplete lineage sorting found in the mitochondria phylogenies and the nuclear-based polytomic phylogenetic structure. Admixture graphs as *qpBrute* can only differentiate in bifurcations, and these three fitting yet different hybridization scenarios could be a mitigation for shared ancestry from an initial polytomy.

We next tested *B. mutus* with *B. sauveli*, *B. javanicus*, and *B. gaurus* (run ID Wild_2 Table S2) and found 59 models that successfully fit the data; each test includes two admixture events, among which any possible pair of species are fitted as admixed species. These results further support a polytomy at the root of species diversification, potentially with significant secondary gene flow. When testing East and South Asian zebu with *B. mutus*, *B. sauveli*, *B. javanicus*, and *B. gaurus*, no model can fit four or less admixture events among >40,000 tested models (Table S2). This ambiguity prevents us from interpreting structured allele sharing between the five populations combined but signals that shared ancestry is ubiquitous.

We lastly tested four subsets of species compositions, involving five populations (Table S2). When testing East and South Asian zebu with *B. sauveli*, *B. javanicus*, and *B. gaurus*, 10 models that fit the data were found, all of which included three admixture events (Figures 3E, 3F, and S4; Table S2). In their deeper structure all models have one major diversification, into a zebu-like main lineage and a wild Asian *Bos* main lineage. However, the lineages consistently mix in the deeper part of the graph (Figures 3E, 3F, and S4). The lack of a sample representing deep ancestral genotypes in the dataset prohibits precise placement and frequency of admixture events. All wild Asian Bos and zebu lineages fit between the extremes of a continuum of the two ancestries, indicating that the polytomic diversification of wild Asian *Bos* involved the acquisition of zebu-like diversity. The continuum extends to the zebu, as we further identify an additional unsampled *Bos* branch, which is incorporated into East Asian zebu. They consistently require ∼10% “exotic” ancestry, inconsistently sourced within the unresolved species network of wild Asian *Bos*, suggesting it derives from a lineage, either partially ancestral to one or several of the lineages leading to *B. sauveli*, *B. javanicus*, and *B. gaurus*, or simply from an individual diversification from the wild Asian *Bos* polytomy.

The speciation scenarios for *B. sauveli*, *B. javanicus*, and *B. gaurus* highlighted in this study are complex, and although each species can be categorized as hybrids, each also forms a clear genetically distinct group, and individuals cluster with members of their respective species. The species represent individually homogenized and drifted lineages from the same prehistoric hybrid swarm. Genera-wide incomplete lineage sorting has been documented across *Ruminantia* (Chen et al., 2019), and the work presented here can now add the *B. sauveli* to the list of ruminants with nuclear genome data to guide interpretation of complex evolution, adding a body of evidence that hybridization is a central feature in mammalian evolution (Fontsere et al., 2019). IUCN lists *B. sauveli* as critically endangered, possibly extinct, with no confirmed sightings for nearly 50 years (Timmins et al., 2016). We hope that wild *B. sauveli* have survived and encourage conservation efforts to protect its potential *refugia* and habitats. In addition, we highlight the potential of researching ecology and evolution of *B. sauveli* based on museum specimens.

### Limitations of the study

Although the data and analyses presented in this study is an advancement of current knowledge in the field, further progress is limited by lack of data. Future work with deeper analyses requires full nuclear, high-coverage and high-quality genomes, from geographically and temporally diverse *B. javanicus, B. gaurus*, and *B. sauveli*.

## STAR★Methods

### Key resources table

**Table:** 

REAGENT or RESOURCE	SOURCE	IDENTIFIER
**Biological samples**		

*B._sauveli*	The Natural History Museum of Denmark	NHMD_231145
*B._sauveli*	The Natural History Museum of Denmark	NHMD_231146
*B._javanicus*	Whipsnade Zoo, UK	DB

**Deposited data**		

*B._javanicus*	Heaton et al. (2016)	KB3281 SRS1620841
*B._javanicus*	Heaton et al. (2016)	OR206 SRS1620842
Bali_cattle	Wu et al. (2018)	ypt2230 SRS2814549
Bali_cattle	Wu et al. (2018)	ypt2227 SRS2814545
Bali_cattle	Wu et al. (2018)	ypt2226 SRS2814550
*B._gaurus*	Heaton et al. (2016)	199911001 SRS1620839
*B._gaurus*	Heaton et al. (2016)	199911002 SRS1620840
*B._gaurus*	Wu et al. (2018)	ypr115 SRS2814552
Gayal	Wu et al. (2018)	223 SRS2814429
Gayal	Wu et al. (2018)	G23 SRS2814433
Gayal	Wu et al. (2018)	ypr153 SRS2814436
*B._mutus*	Qiu et al. (2015)	WYX09 SRS958723
*B._mutus*	Qiu et al. (2015)	WYX17 SRS958726
*B._mutus*	Qiu et al. (2015)	WYX15 SRS958724
Yak	Medugorac et al. (2017)	040 SRS889817
Yak	Heaton et al. (2016)	Queen Allante D171 SRS1620844
Yak	Qiu et al. (2015)	DYS38 SRS958680
*B._bonasus*	Wu et al. (2018)	W_11 SRS2814523
*B._bonasus*	Węcek et al. (2016)	F42_PLANTA SRS1779590
*B._bonasus*	Węcek et al. (2016)	M158_PLATEN SRS1779691
*B._bonasus*	Węcek et al. (2016)	Cc1_8853 SRS1779692
*B._bonasus*	Węcek et al. (2016)	Cc2_22533 SRS1782588
*B._bison*	Wu et al. (2018)	mzc SRS2814553
*B._bison*	Heaton et al. (2016)	199912001 SRS1620843
*B._bison*	Wang et al. (2018)	Bb1 SRS1437873
*B._primigenius*	Park et al. (2015)	Cpc98 SRS1073463
*B._primigenius*	Verdugo et al. (2019)	Ch22 ERS3381593
*B._primigenius*	Verdugo et al. (2019)	Gyu2 ERS3381597
Cattle_Gelbvieh	Stothard et al. (2015)	FREEDOM 178F ET SRS629100
Cattle_Scottish_Highland	Verdugo et al. (2019)	HLAGBRM ERS3381385
Cattle_Suberde (ancient)	Verdugo et al. (2019)	Sub1 ERS3381628
Cattle_Wagyu	Verdugo et al. (2019)	WAGIRLM_01 ERS3381247
Cattle_Mishima	Tsuda et al. (2013)	10031_7821_5 DRS001219
Cattle_Hanwoo	Shin et al. (2014)	A28 SRS457530
Cattle_Lagune	Verdugo et al. (2019)	LAGUNKM_040 ERS3381244
Cattle_Somba	Verdugo et al. (2019)	SOMTGOF_03437 ERS3381386
Cattle_NDama	Kim et al. (2017)	ND719 SRS1512498
Cattle_Hariana	Chen et al. (2018)	Har03 SRS3120723
Cattle_Sahiwal	Chen et al. (2018)	Sha3b SRS3120722
Cattle_Tharparkar	Chen et al. (2018)	Thar1 SRS3120724
Cattle_Brahman	Bickhart et al. (2016)	BIBR1 SRS909351
Cattle_Gir	Bickhart et al. (2016)	BIGI3 SRS909350
Cattle_Nelore	Bickhart et al. (2016)	BINE1 SRS909349
Cattle_Guangfeng	Chen et al. (2018)	JXGF1 SRS2165180
Cattle_Guangfeng	Chen et al. (2018)	JXGF2 SRS2165179
Cattle_Jian	Chen et al. (2018)	JXJA1 SRS2165176
Cattle_Leiqiong	Chen et al. (2018)	HN5M SRS2165085
Cattle_Leiqiong	Chen et al. (2018)	HN12M SRS2165084
Cattle_Wannan	Chen et al. (2018)	WN4M SRS2165094
*S._caffer*	Glanzmann et al. (2016)	98_608 SRS1660476
*B._gaurus*	Hassanin et al. (2012)	JN632604
*B._gaurus*	Rosli et al. (2019)	MK770201
*B._javanicus*	Hassanin et al. (2012)	JN632605
*B._javanicus*	Hassanin et al. (2012)	JN632606
*B._javanicus*	Ishige et al. (2016)	AB915322
Gayal	Prabhu et al. (2019)	MK279401
Gayal	Prabhu et al. (2019)	MK279400
Gayal	Ren et al. (2018)	MF959941
Cattle_I1	Achilli et al. (2009)	FJ971088
Cattle_I1	Achilli et al. (2008)	EU177868
Cattle_I2	Hiendleder et al. (2008)	AF492350
Cattle_I2	Achilli et al. (2008)	EU177870
*B._primigenius*_C	Zhang et al. (2013)	KF525852
Cattle_R	Achilli et al. (2009)	FJ971084
*B._primigenius*_P	Edwards et al. (2010)	GU985279
Cattle_Q	Achilli et al. (2009)	FJ971080
Cattle_T	Achilli et al. (2008)	EU177842
*B. javanicus*	Hassanin and Ropiquet (2007a, 2007b)	EF685913
*B. javanicus*	Galbreath et al. (2006)	DQ459558
*B. javanicus*	Hassanin and Ropiquet (2007a, 2007b)	EF693796
*B. javanicus*	Handschuh and Hassanin (2013)	KF193888
*B. javanicus*	Galbreath et al. (2007)	EF197952
*B. javanicus*	Hassanin and Ropiquet (2007a, 2007b)	EF685912
*B. javanicus*	Hassanin and Ropiquet (2007a, 2007b)	EF685914
*B. javanicus*	Galbreath et al. (2006)	DQ459559
*B. sauveli*	Hassanin and Ropiquet (2004)	AY689189
*B. javanicus*	Hassanin and Ropiquet (2007a, 2007b)	EF693797
*B. javanicus*	Tanaka et al. (1996)	D82889
*B. gaurus*	Hassanin and Ropiquet (2007a, 2007b)	EF685910
*B. gaurus*	Imsoonthornruksa et al. (2012)	GU324988
*B. gaurus*	Direct Submission	FJ190152
*Bubalus bubalis*	Williams et al. (2017)	UMD_CASPUR_WB_2.0
Cattle_Hereford	Rosen et al. (2020)	bostau9

**Critical commercial assays**		

DNeasy Blood & Tissue Kit	QIAGEN	Cat# 69506
MinElute PCR Purification Kit	QIAGEN	Cat# 28006
QIAquick PCR Purification Kit	QIAGEN	Cat# 28106
PfuTurbo Cx Hotstart DNA Polymerase	Agilent	Cat# 600414
AccuPrim Pfx DNA Polymerase	Invitrogen	Cat# 12344024
T4 DNA ligase	New England Biolabs Inc.	Cat# M0202L
T4 Polynucleotide Kinase	New England Biolabs Inc.	Cat# M0201L
T4 DNA Polymerase	New England Biolabs Inc.	Cat# M0203L
BSt 2,0 warmstart polymerase	New England Biolabs Inc.	Cat# M0538L

**Chemicals, peptides, and recombinant proteins**		

Proteinase K	Sigma-Aldrich	Cat#3115844001

**Oligonucleotides**		

Illumina-compatible adapters	Meyer and Kircher (2010)	N/A

**Software and algorithms**		

PALEOMIX pipeline v1.2.13.2	Schubert et al. (2014)	https://github.com/MikkelSchubert/paleomix
AdapterRemoval 2.2.4	Schubert et al. (2016)	https://github.com/MikkelSchubert/adapterremoval
Picard MarkDuplicates v2.18.0	NA	http://broadinstitute.github.io/picard/
GATK v4.1.0.0	McKenna et al. (2010) and DePristo et al. (2011)	https://gatk.broadinstitute.org
ANGSD v0.921	Korneliussen et al. (2014)	https://github.com/ANGSD/angsd
MEGA 10	Kumar et al. (2018)	https://www.megasoftware.net/
MUSCLE	Edgar (2004)	https://www.ebi.ac.uk/Tools/msa/muscle/
SAMtools v1.10	Li et al. (2009)	http://samtools.sourceforge.net/
PCAngsd v0.98	Meisner and Albrechtsen (2018)	https://github.com/Rosemeis/pcangsd
bedtools v2.29.0	Quinlan (2014)	https://github.com/arq5x/bedtools2
RAxML	Stamatakis (2014)	https://sco.h-its.org/exelixis/software.html
ASTRAL-III	Zhang et al. (2018)	https://github.com/smirarab/ASTRAL
Tree Of Life (iTOL) v4	Letunic and Bork (2019)	https://itol.embl.de/
DiscoVista	Sayyari et al. (2018)	https://github.com/esayyari/DiscoVista
qpBrute	Liu et al. (2019)	https://github.com/ekirving/qpbrute
ADMIXTOOLS	Patterson et al. (2012)	https://github.com/DReichLab/AdmixTools

### Resource availability

#### Lead contact

Further information and inquiries about codes, reagents and/or data details may be directed to the lead contact, Mikkel-Holger S. Sinding (mhssinding@gmail.com).

#### Materials availability

This study did not generate new unique reagents.

### Experimental model, and subject details

#### Sample description

The *B. sauveli* specimens NHMD 231145 and NHMD 231145 were collected in Cambodia in 1957 between Phnom Koulen and Koh Ker, where after they have been kept in the collections of the Natural History Museum of Denmark. Both specimens were adult males, NHMD 231145 is an almost intact skull, while NHMD 231146 is a scalp with horns (see Figure S1 for details). For NHMD 231145, data was generated from 6 sup-samples of horn (1), bone (3) and tooth (2), each was a loose fragment in slightly damaged areas, adding no further destructive impact of sampling on the skull. For NHMD 231145, data were generated from 2 sub samples of dried soft tissue, adding no sampling impact on the partial skull itself.

The *B. javanicus* specimen DB is a sample of hair shafts, collected in 1991 in Whipsnade Zoo UK, kept frozen at Smurfit Institute of Genetics, Trinity College Dublin - Ireland. The zoo's records attribute the specimens to a suggested Javan subspecies, indicating a Jarvan origin of the wildtype.

In effort to obtain information on geographic origin of wildtype ancestry of previously published captive *B. gaurus* and *B. javanicus* specimens, we contacted the lead author behind the original publication of the genomes and the zoo’s who managed the specimens. For the *B. gaurus* 199911001 and 199911002 from Omaha's Henry Doorly Zoo - USA, NCBI bioproject PRJNA325061, published by Heaton et al. (2016), no information exists that trace to the geographic origin of wildtype ancestry. The information was obtained by personal communication with Dr. Michael P. Heaton (Genetics, Breeding, and Animal Health Research Unit. USDA, ARS, US Meat Animal Research Center), who have had contact with Omaha's Henry Doorly Zoo about the topic. Interestingly, the closest match for their mitochondrial ancestry can be grouped with a 463bp mitochondrial sequence of a South Indian *B. gaurus* (Figure S2) from Peruvannammuzhi Forest Range in Western Ghats (Kozhikode district, Kerala), indicating potential and/or partial Indian ancestry of the specimens. We note however that larger sample size and more robust data is needed to settle their wildtype ancestry. For the *B. javanicus* specimens KB3281 and OR206 from San Diego Zoo's Beckman Center for Conservation Research - USA, NCBI bioproject PRJNA325061, published by Heaton et al. (2016), the zoo’s records attribute the specimens to a suggested Javan subspecies, indicating a Jarvan origin of the wildtype.

### Method details

#### Ancient DNA extraction, library preparation, and sequencing

*B. sauveli* samples of bone and teeth were digested in a EDTA, urea, proteinase K based buffer as described in Ersmark et al. (2015). *B. sauveli* samples of horn and dried soft tissue were digested in a DTT, proteinase K based buffer as described in Gilbert et al. (2007). These individual digests of bone, teeth, horn and dried soft tissue were purified as described in Dabney et al. (2013), however using a modified binding buffer as in Allentoft et al. (2015). DNA from a *B. javanicus* sample of hair shafts were extracted using the DNeasy Blood & Tissue Kit (QIAGEN, Hilden, Germany) following the manufacturer’s protocol. Purified DNA extracts of *B. sauveli* were incorporated into libraries following the single tube protocol (Carøe et al., 2017) with the reaction setup modifications of Mak et al. (2017). Specimen B._sauveli_NHMD_231145 was sequenced on one lane of BGISEQ-500 - SR100, as well as 55% of a lane of Illumina HighSeq 2500 - SR80. Specimen B._sauveli_NHMD_231146 was sequenced on two lanes of BGISEQ-500 - SR100. The *B. javanicus* DNA extract was incorporated into a sequencing library following (Meyer and Kircher, 2010) and sequenced on one lane of Illumina HighSeq 2500 - SR100. Details on sequencing output is given in Table S1, sheet Mapping Stats.

### Quantification and statistical analysis

#### Data processing pipeline

All newly generated and previously published raw read data (see Key resources table - deposited data) was processed in a PALEOMIX pipeline v1.2.13.2 (Schubert et al., 2014). Low quality and missing bases were trimmed from the reads with default settings, and adaptors, dimers and sequences of less than 25bp were removed using AdapterRemoval 2.2.4 (Schubert et al., 2016). To avoid biases associated with mapping to an ingroup (Gopalakrishnan et al., 2017) in downstream ancestry analyses, retained reads were mapped against scaffolds above 50.000bp of the outgroup - de novo Water buffalo (*Bubalus bubalis*) UMD_CASPUR_WB_2.0 (Williams et al., 2017). In addition, to generate mitochondrial genome sequences against a taurine cattle reference, non-cattle specimens were further mapped to the Hereford bostau9 (Rosen et al., 2020) using the same pipeline as above. Using the full genome and focusing only on reads mapping to the mitochondrial genome were performed to reduce potential nunts in the mitochondrial assembly (Castruita et al., 2015). Alignments were made using bwa-aln v0.7.16a algorithm (Li and Durbin, 2009) including minimum base mapping quality to optimize initial coverage. Filters targeting mapping and base quality were added at a later stage as appropriate in the specific analysis. Next, Picard MarkDuplicates v2.18.0 (http://broadinstitute.github.io/picard/) was used to filter for PCR and optical duplicates. Finally, GATK v4.1.0.0 (McKenna et al., 2010; DePristo et al., 2011) was used to perform the indel realignment step with no external indel database.

#### Mitochondrial DNA analysis

Mitochondrial genome assemblies for *B. sauveli, B. gaurus*, gayals, *B. javanicus* and Bali cattle, were obtained by mapping raw data to both nuclear and mtDNA taurine cattle reference genome bostau9 (Rosen et al., 2020), mapping to the full genome to remove potential numts (nuclear mitochondrial DNAs) from the assembly (Castruita et al., 2015). The taurine cattle mitochondria is phylogenetically closer to *Bos* than to the water buffalo (Hassanin et al., 2012), intuitively improving mapping quality. Using ANGSD v0.921(Korneliussen et al., 2014), sites with a minimal coverage of 3 were called in the mitochondrial scaffold of the bam files generated using bostau9 (Rosen et al., 2020) (see Data processing pipeline) and exported as individual files. All mitochondrial trees were generated using the same settings in MEGA 10 (Kumar et al., 2018), mitochondrial sequences were aligned by UPGMB clustering using MUSCLE (Edgar, 2004), and modelled as neighbour joining (NJ) trees using 500 bootstrap replications and complete deletion of missing sites. Sites with a minimum depth of 3x ranging from 99% to 36% of the individual mitochondrial genomes, were aligned to publicly available mitochondrial genomes (see Key resources table - deposited data) from diverse *Bos*, allowing for a 3657bp overlap of coverage across all samples. The alignment was first used to generate a neighbor-joining (NJ) tree (Figures 1B and S2), including selected *Bos* specimens for which we have full nuclear genome data. Second, we generated a NJ tree focusing on 1140 bp of the cytochrome b region (Cytb), including the *B. sauveli* holotype (Figure S2). And third, we produced a NJ tree including only full mitochondrial genomes and the near complete *B. sauveli* mitochondrial genomes (Figure S2). The newly generated sequences disperse across the trees according to their respective species and geography, showing that the bostau9 mitochondrial scaffold is a useful reference for mapping other *Bos* mitochondrial genomes. The Cytb tree mirrors the full mitochondria phylogenetic reconstruction, indicating Cytb is a useful proxy to recover the overall mitochondrial structure of Asian *Bos*. However, the full mitochondrial genomes provide better support and likely increased fine-structure resolution.

#### Principal component analysis

Genotypes were called from genotype likelihoods with ANGSD v0.921 (Korneliussen et al., 2014), to avoid biases resulting from genotype calling in low coverage samples (Nielsen et al., 2011). The ANGSD processing used SAMtools v1.10 (Li et al., 2009) formula (-GL 1), discarding bases with base qualities lower than 20 (-minQ 20), and reads with mapping quality lower than 20 (-minmapq 20), as well as sites with minor allele frequencies below 0.1 (-minMaf 0.1). Analysis was restricted to sites covered in minimally 95% of the individuals in the specific dataset, and transition sites were discarded (-rmTrans 1) in order to minimize aDNA damage included in the ancient samples (Briggs et al., 2007). The genotype likelihoods were interpreted as PCA covariance matrices using PCAngsd v0.98 (Meisner and Albrechtsen, 2018), which subsequently was visualised using Rstudio (Team, 2020).

#### ASTRAL-III analysis

A nuclear genome phylogeny was generated based on 1000 individual trees, each built on 1000 randomly selected sequences of 5000bp. The initial sequences were selected using bedtools v2.29.0 (Quinlan, 2014) *random* (-l 5000 and -n 1000), generated as a consensus (-dofasta 2) sequence using ANGSD v0.921 (Korneliussen et al., 2014), including sites of minimally 3x (-setminDepthInd 3) and discarding bases with base quality and mapping quality lower than 20 (-minQ 20 -minmapq 20). Individual trees for each region were generated using RAxML (Stamatakis, 2014), concatenated together to generate a single species tree using ASTRAL-III (Zhang et al., 2018) with default parameters. The phylogeny was visualised using the online tool Interactive Tree Of Life (iTOL) v4 (Letunic and Bork, 2019).

#### DiscoVista analysis

To evaluate the support of alternative topologies in the Astral tree, DiscoVista (Sayyari et al., 2018) was used to visualize the discordance between the 1000 gene trees and the species tree. Samples belonging to the same species were collapsed together using an annotation file (-a option) and the African buffalo (*Syncerus caffer*) was specified as outgroup (option -g). *T*he relative frequencies of gene trees supporting specific species topologies were determined.

#### D-statistics

D-statistics (ABBA-BABA) were used to investigate allele sharing between genomes by using ANGSD v0.921 (Korneliussen et al., 2014) (-doAbbababa 1), which subsequently was visualised using Rstudio. Analysis was restricted to sites with base quality and mapping quality above 20 (-minQ 20 -minmapq 20), transversions (-rmTrans 1), using an African buffalo (*S. caffer*) as outgroup and applying the following parameters: -doCounts 1 -useLast 1 -blockSize 1000000. Only D-statistics with a Z-score above 3 and below 3 were considered significant.

#### qpBrute analysis

We created a haploid dataset consisting of all Bison and Bos genomes included in the study (Table 1) and a *Syncerus caffer* used as the outgroup. For each sample at each genomic site, we sampled a random read using ANGSD v0.921 (Korneliussen et al., 2014) from the reads with a minimum mapping quality of 30 and bases with minimum quality of 20. Transitions were discarded in order to reduce the aDNA derived error in the historical samples, as well as scaffolds shorter than 1 Mb. The final dataset consisted of 3,718,284 transversion sites. We performed an heuristic search of the graph space using qpBrute (Liu et al., 2019) which is a python based algorithm that employs qpGraph from the ADMIXTOOLS package (Patterson et al., 2012) to fit complex admixture models in a stepwise fashion. Starting from the root, at each iteration the program adds a new leaf to the graph until it exhausts the list of populations included in that particular run. If a node cannot be inserted either directly on a branch or as the product of an admixture event without creating f4 outliers, the sub-graph is discarded. For each run, the populations included, represent merged clusters of *Bos* individuals rooted to the African buffalo (*S. caffer*), for sample and run combinations see Table S1.

## Data Availability

This study did not generate any unpublished custom code, software, or algorithm. All newly generated raw reads have been uploaded to NCBI in the following bioproject PRJNA764745.

## Supplemental information

Document S1. Figures S1–S4

Table S1. Details on samples, data and read mapping statistics, related to “Main Table 1”

Table S2. Details on qpBrute models, data and outputs, related to “Main Figure 3”

## References

[bib1] Achilli A., Bonfiglio S., Olivieri A., Malusà A., Pala M., Hooshiar Kashani B., Perego U.A., Ajmone-Marsan P., Liotta L., Semino O. (2009). The multifaceted origin of taurine cattle reflected by the mitochondrial genome. PLoS One.

[bib2] Achilli A., Olivieri A., Pellecchia M., Uboldi C., Colli L., Al-Zahery N., Accetturo M., Pala M., Hooshiar Kashani B., Perego U.A. (2008). Mitochondrial genomes of extinct aurochs survive in domestic cattle. Curr. Biol..

[bib3] Allentoft M.E., Sikora M., Sjögren K.-G., Rasmussen S., Rasmussen M., Stenderup J., Damgaard P.B., Schroeder H., Ahlström T., Vinner L. (2015). Population genomics of Bronze Age Eurasia. Nature.

[bib4] Allman E.S., Degnan J.H., Rhodes J.A. (2011). Identifying the rooted species tree from the distribution of unrooted gene trees under the coalescent. J. Math. Biol..

[bib5] Bickhart D.M., Xu L., Hutchison J.L., Cole J.B., Null D.J., Schroeder S.G., Song J., Garcia J.F., Sonstegard T.S., Van Tassell C.P. (2016). Diversity and population-genetic properties of copy number variations and multicopy genes in cattle. DNA Res..

[bib6] Bohlken H. (1963). Bemerkungen zu drei Schädeln des kouprey, Bos(Bibos) sauveli Urbain, im Pariser Museum. Zool. Anz..

[bib7] Bohlken H. (1961). Der kouprey, Bos (Bibos) sauveli Urbain 1937. Z. Sauegetierkunde.

[bib8] Bohlken H. (1958). Vergleichende untersuchungen an wildrindern (Tribus bovini Simpson, 1945). Zool. Jahrb..

[bib9] Briggs A.W., Stenzel U., Johnson P.L.F., Green R.E., Kelso J., Prüfer K., Meyer M., Krause J., Ronan M.T., Lachmann M., Pääbo S. (2007). Patterns of damage in genomic DNA sequences from a Neandertal. Proc. Natl. Acad. Sci. U. S. A..

[bib10] Carøe C., Gopalakrishnan S., Vinner L., Mak S.S.T., Sinding M.-H.S., Samaniego J.A., Wales N., Sicheritz-Pontén T., Gilbert M.T.P. (2017). Single-tube library preparation for degraded DNA. Methods Ecol. Evol..

[bib11] Castruita J.A.S., Mendoza M.L.Z., Barnett R., Wales N., Gilbert M.T.P. (2015). Odintifier-A computational method for identifying insertions of organellar origin from modern and ancient high-throughput sequencing data based on haplotype phasing. BMC Bioinformatics.

[bib12] Chen L., Qiu Q., Jiang Y., Wang K., Lin Z., Li Z., Bibi F., Yang Y., Wang J., Nie W. (2019). Large-scale ruminant genome sequencing provides insights into their evolution and distinct traits. Science.

[bib13] Chen N., Cai Y., Chen Q., Li R., Wang K., Huang Y., Hu S., Huang S., Zhang H., Zheng Z. (2018). Whole-genome resequencing reveals world-wide ancestry and adaptive introgression events of domesticated cattle in East Asia. Nat. Commun..

[bib14] Chen S., Lin B.-Z., Baig M., Mitra B., Lopes R.J., Santos A.M., Magee D.A., Azevedo M., Tarroso P., Sasazaki S. (2010). Zebu cattle are an exclusive legacy of the South Asia neolithic. Mol. Biol. Evol..

[bib15] Coolidge H.J. (1940). Memoirs of the Museum of Comparative Zoology at Harvard College.

[bib16] Dabney J., Knapp M., Glocke I., Gansauge M.-T., Weihmann A., Nickel B., Valdiosera C., García N., Pääbo S., Arsuaga J.-L., Meyer M. (2013). Complete mitochondrial genome sequence of a Middle Pleistocene cave bear reconstructed from ultrashort DNA fragments. Proc. Natl. Acad. Sci. U. S. A..

[bib17] DePristo M.A., Banks E., Poplin R., Garimella K.V., Maguire J.R., Hartl C., Philippakis A.A., del Angel G., Rivas M.A., Hanna M. (2011). A framework for variation discovery and genotyping using next-generation DNA sequencing data. Nat. Genet..

[bib18] Duckworth, J.W., Sankar, K., Williams, A.C., Samba Kumar, N., Timmins, R.J., 2016. Bos gaurus. The IUCN Red List of Threatened Species 2016 e.T2891A46363646, Downloaded on 21 July 2020.

[bib19] Edgar R.C. (2004). MUSCLE: multiple sequence alignment with high accuracy and high throughput. Nucleic Acids Res..

[bib20] Edmond-Blanc F. (1947). A Contribution to the Knowledge of the Cambodian wild ox or Kouproh. J. Mammal..

[bib21] Edwards C.J., Magee D.A., Park S.D.E., McGettigan P.A., Lohan A.J., Murphy A., Finlay E.K., Shapiro B., Chamberlain A.T., Richards M.B. (2010). A complete mitochondrial genome sequence from a mesolithic wild aurochs (Bos primigenius). PLoS One.

[bib22] Ersmark E., Orlando L., Sandoval-Castellanos E., Barnes I., Barnett R., Stuart A., Lister A., Dalén L. (2015). Population demography and genetic diversity in the Pleistocene cave lion. Open Quat..

[bib23] Fontsere C., de Manuel M., Marques-Bonet T., Kuhlwilm M. (2019). Admixture in mammals and how to understand its functional implications: on the abundance of gene flow in mammalian species, its impact on the genome, and roads into a functional understanding: on the abundance of gene flow in mammalian species, its impact on the genome, and roads into a functional understanding. Bioessays.

[bib24] Galbreath G.J., Mordacq J.C., Weiler F.H. (2007). An evolutionary conundrum involving kouprey and banteng: a response from Galbreath, Mordacq and Weiler. J. Zool..

[bib25] Galbreath G.J., Mordacq J.C., Weiler F.H. (2006). Genetically solving a zoological mystery: was the kouprey (Bos sauveli) a feral hybrid?. J. Zool..

[bib26] Gardner, P., Hedges, S., Pudyatmoko, S., Gray, T.N.E., Timmins, R.J., 2016. Bos javanicus. The IUCN Red List of Threatened Species 2016 e.T2888A46362970, Downloaded on 21 July 2020.

[bib27] Geraads D. (1992). Phylogenetic analysis of the tribe bovini (Mammalia: Artiodactyla). Zool. J. Linn. Soc..

[bib28] Gilbert M.T.P., Tomsho L.P., Rendulic S., Packard M., Drautz D.I., Sher A., Tikhonov A., Dalén L., Kuznetsova T., Kosintsev P. (2007). Whole-genome shotgun sequencing of mitochondria from ancient hair shafts. Science.

[bib29] Glanzmann B., Möller M., le Roex N., Tromp G., Hoal E.G., van Helden P.D. (2016). The complete genome sequence of the African buffalo (Syncerus caffer). BMC Genomics.

[bib30] Gopalakrishnan S., Castruita J.S., Sinding M.H.S., Kuderna L., Räikkönen J., Petersen B., SIcheritz-Ponten T., Larson G., Orlando L., Marques-Bonet T. (2017). The wolf reference genome sequence (Canis lupus lupus) and its implications for Canis spp. population genomics. BMC Genomics.

[bib31] Gower G., Chen K., Richards S.M., Llamas B., Mitchell K.J., Ho S.Y.W., Kosintsev P., Lee M.S.Y., Baryshnikov G., Bollongino R. (2016). Early cave art and ancient DNA record the origin of European bison. Nat. Commun..

[bib32] Grange T., Brugal J.-P., Flori L., Gautier M., Uzunidis A., Geigl E.-M. (2018). The evolution and population diversity of Bison in Pleistocene and Holocene Eurasia: Sex Matters. Diversity.

[bib33] Groves C.P. (1981). Systematic relationships in the bovini (Artiodactyla, Bovidae). J. Zoolog. Syst. Evol. Res..

[bib34] Handschuh M., Hassanin A. (2013). Pure banteng Bos javanicus persist in southern Preah Vihear province, central Cambodia, despite apparent hybridisation with domestic cattle. Nat. Hist. Bull. Siam Soc..

[bib35] Hassanin A., Delsuc F., Ropiquet A., Hammer C., Jansen van Vuuren B., Matthee C., Ruiz-Garcia M., Catzeflis F., Areskoug V., Nguyen T.T., Couloux A. (2012). Pattern and timing of diversification of Cetartiodactyla (Mammalia, Laurasiatheria), as revealed by a comprehensive analysis of mitochondrial genomes. C. R. Biol..

[bib36] Hassanin A., Ropiquet A. (2007). What is the taxonomic status of the Cambodian banteng and does it have close genetic links with the kouprey?. J. Zool..

[bib37] Hassanin A., Ropiquet A. (2007). Resolving a zoological mystery: the kouprey is a real species. Proc. Biol. Sci..

[bib38] Hassanin A., Ropiquet A. (2004). Molecular phylogeny of the tribe bovini (Bovidae, Bovinae) and the taxonomic status of the kouprey, Bos sauveli Urbain 1937. Mol. Phylogenet. Evol..

[bib39] Heaton M.P., Smith T.P.L., Carnahan J.K., Basnayake V., Qiu J., Simpson B., Kalbfleisch T.S. (2016). P6026 Using diverse US beef cattle genomes to identify missense mutations in EPAS1, a gene associated with high-altitude pulmonary hypertension. J. Anim. Sci..

[bib40] Hedges S., Groves C.P., Duckworth J.W., Meijaard E., Timmins R.J., Burton J.A. (2007). Was the kouprey a feral hybrid? A response to Galbreath et al. (2006). J. Zool..

[bib41] Hiendleder S., Lewalski H., Janke A. (2008). Complete mitochondrial genomes of Bos taurus and Bos indicus provide new insights into intra-species variation, taxonomy and domestication. Cytogenet. Genome Res..

[bib42] Higham C. (1996).

[bib43] Imsoonthornruksa S., Srirattana K., Phewsoi W., Tunwattana W., Parnpai R., Ketudat-Cairns M. (2012). Segregation of donor cell mitochondrial DNA in gaur–bovine interspecies somatic cell nuclear transfer embryos, fetuses and an offspring. Mitochondrion.

[bib44] Ishige T., Gakuhari T., Hanzawa K., Kono T., Sunjoto I., Sukor J.R.A., Ahmad A.H., Matsubayashi H. (2016). Complete mitochondrial genomes of the tooth of a poached Bornean banteng (Bos javanicus lowi; Cetartiodactyla, Bovidae). Mitochondrial DNA A. DNA Mapp. Seq. Anal.

[bib45] Kim J., Hanotte O., Mwai O.A., Dessie T., Bashir S., Diallo B., Agaba M., Kim K., Kwak W., Sung S. (2017). The genome landscape of indigenous African cattle. Genome Biol..

[bib46] Korneliussen T.S., Albrechtsen A., Nielsen R. (2014). ANGSD: analysis of next generation sequencing data. BMC Bioinformatics.

[bib47] Kumar S., Stecher G., Li M., Knyaz C., Tamura K. (2018). Mega X: Molecular evolutionary Genetics analysis across Computing platforms. Mol. Biol. Evol..

[bib48] Letunic I., Bork P. (2019). Interactive Tree of Life (iTOL) v4: recent updates and new developments. Nucleic Acids Res..

[bib49] Li H., Durbin R. (2009). Fast and accurate short read alignment with Burrows–Wheeler transform. Bioinformatics.

[bib50] Li H., Handsaker B., Wysoker A., Fennell T., Ruan J., Homer N., Marth G., Abecasis G., Durbin R., 1000 Genome Project data Processing Subgroup (2009). The sequence alignment/Map format and SAMtools. Bioinformatics.

[bib51] Liu L., Bosse M., Megens H.-J., Frantz L.A.F., Lee Y.-L., Irving-Pease E.K., Narayan G., Groenen M.A.M., Madsen O. (2019). Genomic analysis on pygmy hog reveals extensive interbreeding during wild boar expansion. Nat. Commun..

[bib52] MacHugh D.E. (1996).

[bib53] Mak S.S.T., Gopalakrishnan S., Carøe C., Geng C., Liu S., Sinding M.-H.S., Kuderna L., Zhang W., Fu S., Vieira F. (2017). Comparative performance of the BGISEQ-500 vs Illumina sequencing platforms for palaeogenomic sequencing. Gigascience.

[bib54] McKenna A., Hanna M., Banks E., Sivachenko A., Cibulskis K., Kernytsky A., Garimella K., Altshuler D., Gabriel S., Daly M., DePristo M.A. (2010). The Genome Analysis Toolkit: a MapReduce framework for analyzing next-generation DNA sequencing data. Genome Res..

[bib55] Medugorac I., Graf A., Grohs C., Rothammer S., Zagdsuren Y., Gladyr E., Zinovieva N., Barbieri J., Seichter D., Russ I. (2017). Whole-genome analysis of introgressive hybridization and characterization of the bovine legacy of Mongolian yaks. Nat. Genet..

[bib56] Meisner J., Albrechtsen A. (2018). Inferring population structure and admixture proportions in low-depth NGS data. Genetics.

[bib57] Meyer M., Kircher M. (2010). Illumina sequencing library preparation for highly multiplexed target capture and sequencing. Cold Spring Harb. Protoc..

[bib58] Nielsen R., Paul J.S., Albrechtsen A., Song Y.S. (2011). Genotype and SNP calling from next-generation sequencing data. Nat. Rev. Genet..

[bib59] Orlando L., Ginolhac A., Zhang G., Froese D., Albrechtsen A., Stiller M., Schubert M., Cappellini E., Petersen B., Moltke I. (2013). Recalibrating Equus evolution using the genome sequence of an early Middle Pleistocene horse. Nature.

[bib60] Park S.D.E., Magee D.A., McGettigan P.A., Teasdale M.D., Edwards C.J., Lohan A.J., Murphy A., Braud M., Donoghue M.T., Liu Y. (2015). Genome sequencing of the extinct Eurasian wild aurochs, Bos primigenius, illuminates the phylogeography and evolution of cattle. Genome Biol..

[bib61] Patterson N., Moorjani P., Luo Y., Mallick S., Rohland N., Zhan Y., Genschoreck T., Webster T., Reich D. (2012). Ancient admixture in human history. Genetics.

[bib62] Pfeffer P., Kim-San O. (1967). Le kouprey, Bos (Bibos) sauveli Urbain, 1937; Discussion systématique et statut actuel. Hypothèse sur l’origine du zébu (Bos indicus). Mammalia.

[bib63] Prabhu V.R., Arjun M.S., Bhavana K., Kamalakkannan R., Nagarajan M. (2019). Complete mitochondrial genome of Indian mithun, Bos frontalis and its phylogenetic implications. Mol. Biol. Rep..

[bib64] Qiu Q., Wang L., Wang K., Yang Y., Ma T., Wang Z., Zhang X., Ni Z., Hou F., Long R. (2015). Yak whole-genome resequencing reveals domestication signatures and prehistoric population expansions. Nat. Commun..

[bib65] Quinlan A.R. (2014). BEDTools: the Swiss-Army tool for genome Feature analysis. Curr. Protoc. Bioinformatics.

[bib66] Ren Q., Liu Y., Xie X., Yan B., Zhang K., Yang Y., Qiu Q. (2018). Complete mitochondrial genome of bovine species Gayal (Bos frontalis). Conserv. Genet. Resour..

[bib67] Rosen B.D., Bickhart D.M., Schnabel R.D., Koren S., Elsik C.G., Tseng E., Rowan T.N., Low W.Y., Zimin A., Couldrey C. (2020). De novo assembly of the cattle reference genome with single-molecule sequencing. Gigascience.

[bib68] Rosli N., Sitam F.T., Rovie-Ryan J.J., Gan H.M., Lee Y.P., Ithnin Hartini, Gani Millawati, Md-Zain B.M., Abdullah M.T., Mohd Firdaus Ariff Abdul Razak (2019). The complete mitochondrial genome of Malayan Gaur (Bos gaurus hubbacki) from Peninsular Malaysia. Mitochondrial DNA B.

[bib69] Saijuntha W., Petney T., Kongbuntad W. (2013). Genetic characterization of banteng (Bos javanicus) in Lam Pao Wildlife Conservation Development and Promotion Station, Kalasin province. Genomics Genet..

[bib70] Sayyari E., Whitfield J.B., Mirarab S. (2018). DiscoVista: Interpretable visualizations of gene tree discordance. Mol. Phylogenet. Evol..

[bib71] Schubert M., Ermini L., Der Sarkissian C., Jónsson H., Ginolhac A., Schaefer R., Martin M.D., Fernández R., Kircher M., McCue M. (2014). Characterization of ancient and modern genomes by SNP detection and phylogenomic and metagenomic analysis using PALEOMIX. Nat. Protoc..

[bib72] Schubert M., Lindgreen S., Orlando L. (2016). AdapterRemoval v2: rapid adapter trimming, identification, and read merging. BMC Res. Notes.

[bib73] Shin D.-H., Lee H.-J., Cho S., Kim H.J., Hwang J.Y., Lee C.-K., Jeong J., Yoon D., Kim H. (2014). Deleted copy number variation of Hanwoo and Holstein using next generation sequencing at the population level. BMC Genomics.

[bib74] Stamatakis A. (2014). RAxML version 8: a tool for phylogenetic analysis and post-analysis of large phylogenies. Bioinformatics.

[bib75] Stothard P., Liao X., Arantes A.S., De Pauw M., Coros C., Plastow G.S., Sargolzaei M., Crowley J.J., Basarab J.A., Schenkel F. (2015). A large and diverse collection of bovine genome sequences from the Canadian Cattle Genome Project. GigaScience.

[bib76] Tanaka K., Solis C.D., Masangkay J.S., Maeda K.-I., Kawamoto Y., Namikawa T. (1996). Phylogenetic relationship among all living species of the genusBubalus based on DNA sequences of the cytochromeb gene. Biochem. Genet..

[bib77] Team R. (2020).

[bib78] Timmins, R.J., Burton, J., Hedges, S., 2016. Bos sauveli. The IUCN Red List of Threatened Species 2016 e.T2890A46363360, Downloaded on 22 July 2020.

[bib79] Tsuda K., Kawahara-Miki R., Sano S., Imai M., Noguchi T., Inayoshi Y., Kono T. (2013). Abundant sequence divergence in the native Japanese cattle Mishima-Ushi (Bos taurus) detected using whole-genome sequencing. Genomics.

[bib80] Urbain A. (1937). Le Kou Prey ou boeuf gris cambodgien. Bull. Soc. Zool. Fr..

[bib81] Verdugo M.P., Mullin V.E., Scheu A., Mattiangeli V., Daly K.G., Maisano Delser P., Hare A.J., Burger J., Collins M.J., Kehati R. (2019). Ancient cattle genomics, origins, and rapid turnover in the Fertile Crescent. Science.

[bib82] Vithayanon C., Bhumpakphan N. (2004). Historical distribution range of the Kouprey from three fossil locality sites in north Northeastern Thailand. J. Wildl. Thailand.

[bib83] Wang K., Lenstra J.A., Liu L., Hu Q., Ma T., Qiu Q., Liu J. (2018). Incomplete lineage sorting rather than hybridization explains the inconsistent phylogeny of the wisent. Commun. Biol..

[bib84] Wangkumhang P., Wilantho A., Shaw P.J., Flori L., Moazami-Goudarzi K., Gautier M., Duangjinda M., Assawamakin A., Tongsima S. (2015). Genetic analysis of Thai cattle reveals a Southeast Asian indicine ancestry. PeerJ.

[bib85] Węcek K., Hartmann S., Paijmans J.L.A., Taron U., Xenikoudakis G., Cahill J.A., Heintzman P.D., Shapiro B., Baryshnikov G., Bunevich A.N. (2016). Complex admixture Preceded and Followed the Extinction of wisent in the wild. Mol. Biol. Evol..

[bib86] Wharton C.H. (1957).

[bib87] Williams J.L., Iamartino D., Pruitt K.D., Sonstegard T., Smith T.P.L., Low W.Y., Biagini T., Bomba L., Capomaccio S., Castiglioni B. (2017).

[bib88] Wu D.-D., Ding X.-D., Wang S., Wójcik J.M., Zhang Y., Tokarska M., Li Y., Wang M.-S., Faruque O., Nielsen R. (2018). Pervasive introgression facilitated domestication and adaptation in the Bos species complex. Nat. Ecol. Evol..

[bib89] Zhang C., Rabiee M., Sayyari E., Mirarab S. (2018). ASTRAL-III: polynomial time species tree reconstruction from partially resolved gene trees. BMC Bioinformatics.

[bib90] Zhang H., Paijmans J.L.A., Chang F., Wu X., Chen G., Lei C., Yang X., Wei Z., Bradley D.G., Orlando L. (2013). Morphological and genetic evidence for early Holocene cattle management in northeastern China. Nat. Commun..

