# Supplementary material for: Kouprey (Bos sauveli) genomes unveil polytomic origin of wild Asian Bos
Source: iScience. 2021 Oct 6;24(11):103226. doi: 10.1016/j.isci.2021.103226 (PMC8531564; doi:10.1016/j.isci.2021.103226)
Supplement: Document S1. Figures S1–S4 [file mmc1.pdf]

iScience, Volume *24*

Supplemental information

Kouprey (*Bos sauveli*) genomes unveil polytomic origin of wild Asian *Bos*

Mikkel-Holger S. Sinding, Marta M. Ciucani, Jazmín Ramos-Madrigal, Alberto Carmagnini, Jacob Agerbo Rasmussen, Shaohong Feng, Guangji Chen, Filipe G. Vieira, Valeria Mattiangeli, Rajinder K. Ganjoo, Greger Larson, Thomas Sicheritz-Pontén, Bent Petersen, Laurent Frantz, M. Thomas P. Gilbert, and Daniel G. Bradley

Supplemental figures

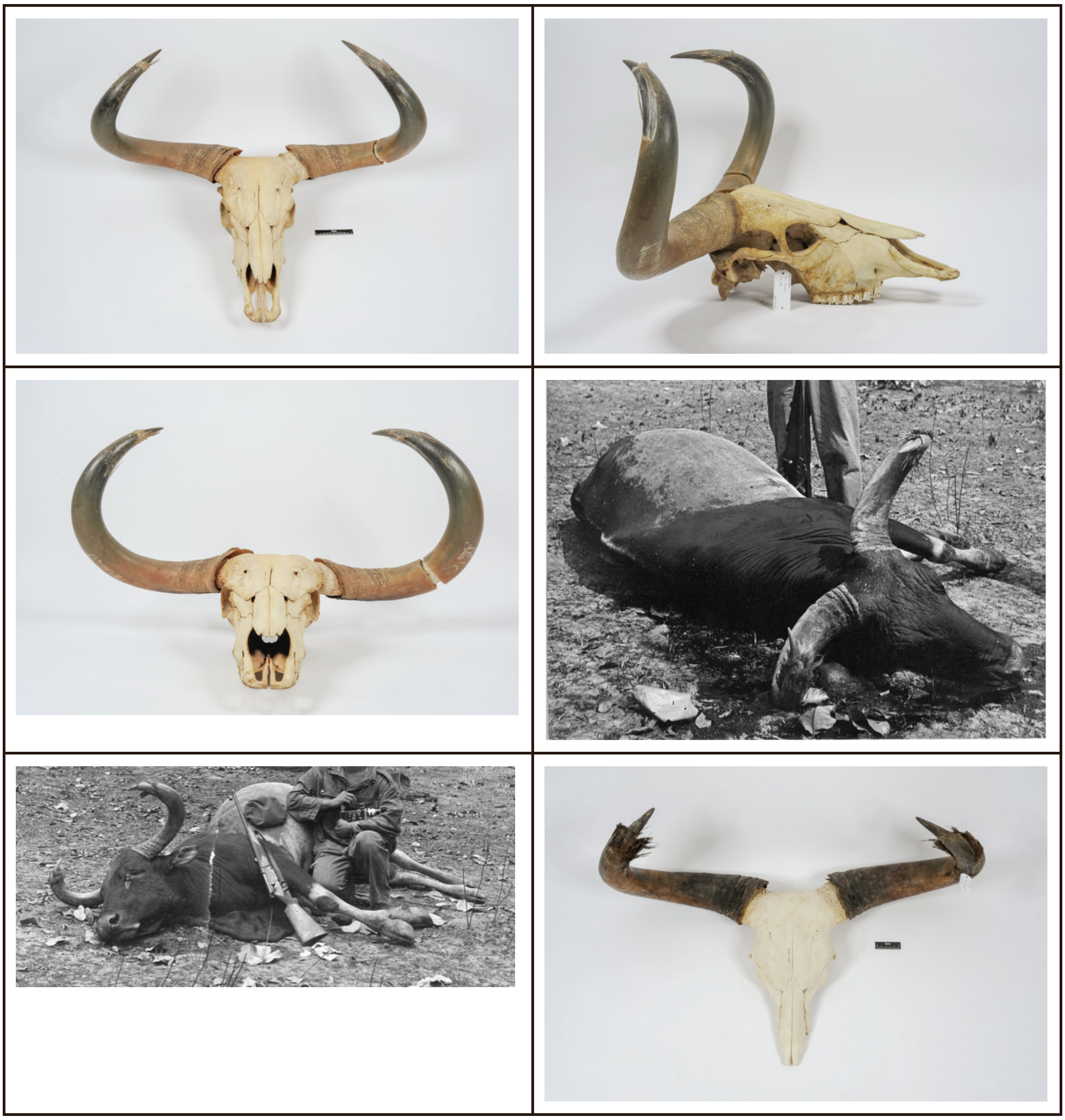

Figure S1. ***Bos sauveli*** **specimens.**

The photos in the top and middle-left are specimen NHMD 231145. The photos middle-right and bottom are NHMD 231146. A centimetre-scale is included in a photo of each skull. Related to ”Experimental Model and Subject Details - Sample description”.

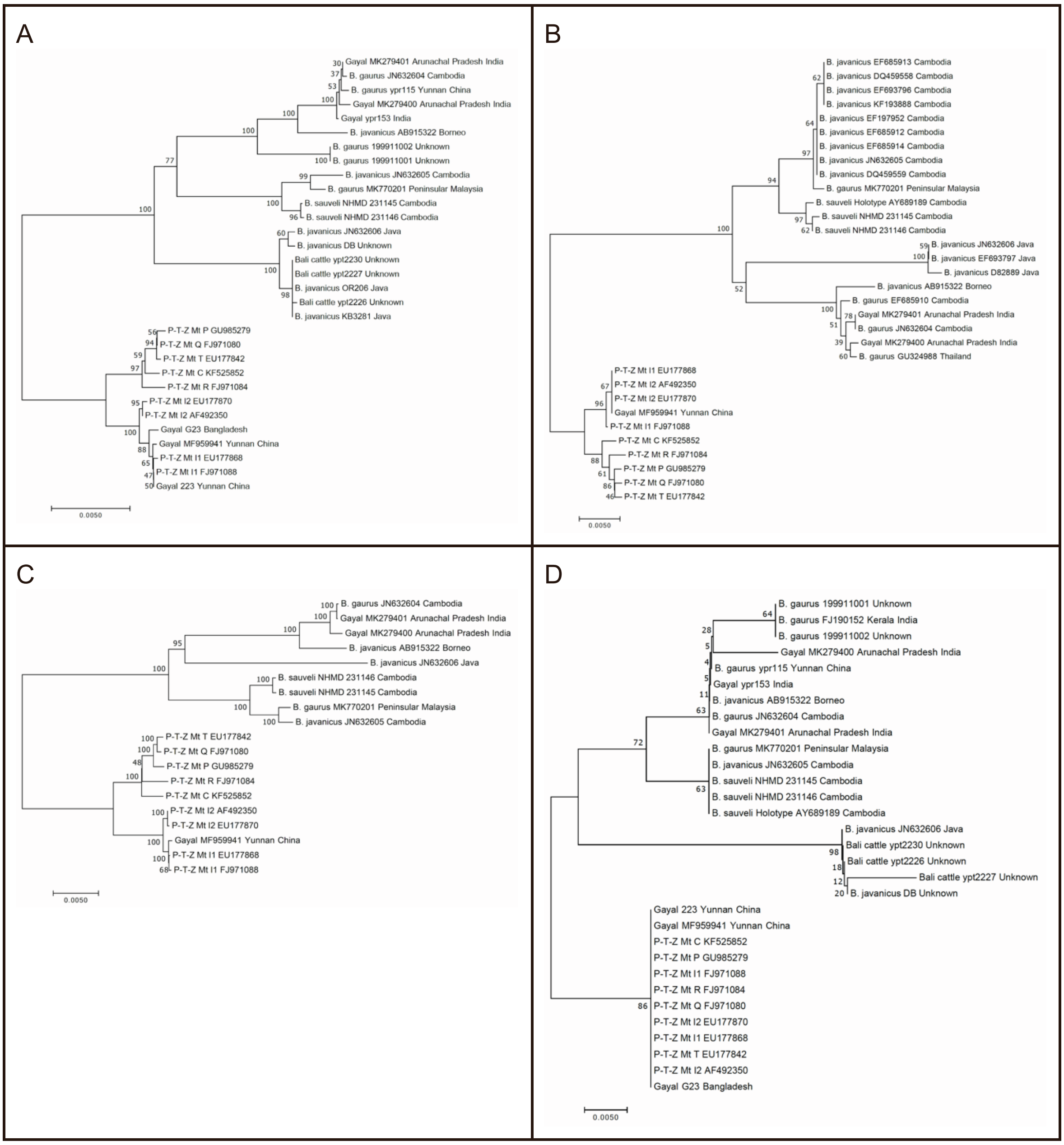

Figure S2. Additional mitochondrial phylogenies.

A Uncollapsed mitochondrial phylogeny used in main figure 1. Neighbor joining tree based on 31 specimens, with sites across the full mitochondrial genome, with a total 3657bp overlap of coverage across all samples. **B** Mitochondrial phylogeny including cytB specific specimens. Neighbor joining tree based on 32 specimens, with sites across a 1140bp mitochondrial cytB region, with a total 861bp overlap of coverage across all samples. Dataset included additional *B. javanicus*, *B. gaurus* and the Holotype of *B. sauveli*. **C** Mitochondrial phylogeny using near-complete genomes. Neighbor joining tree based on 19 specimens, with sites across the full mitochondrial genome, with a total 12060bp overlap of coverage across all samples. **D** Mitochondrial cytB phylogeny including an Indian *B. gaurus* sequence. Neighbor joining tree based on 31 specimens, with sites across a 463bp mitochondrial cytB region, with a total 138bp overlap of coverage across all samples. The nodes labels represent the percentage of bootstrap support for each clade computed using 500 replicates. Related to "Main figure 1".

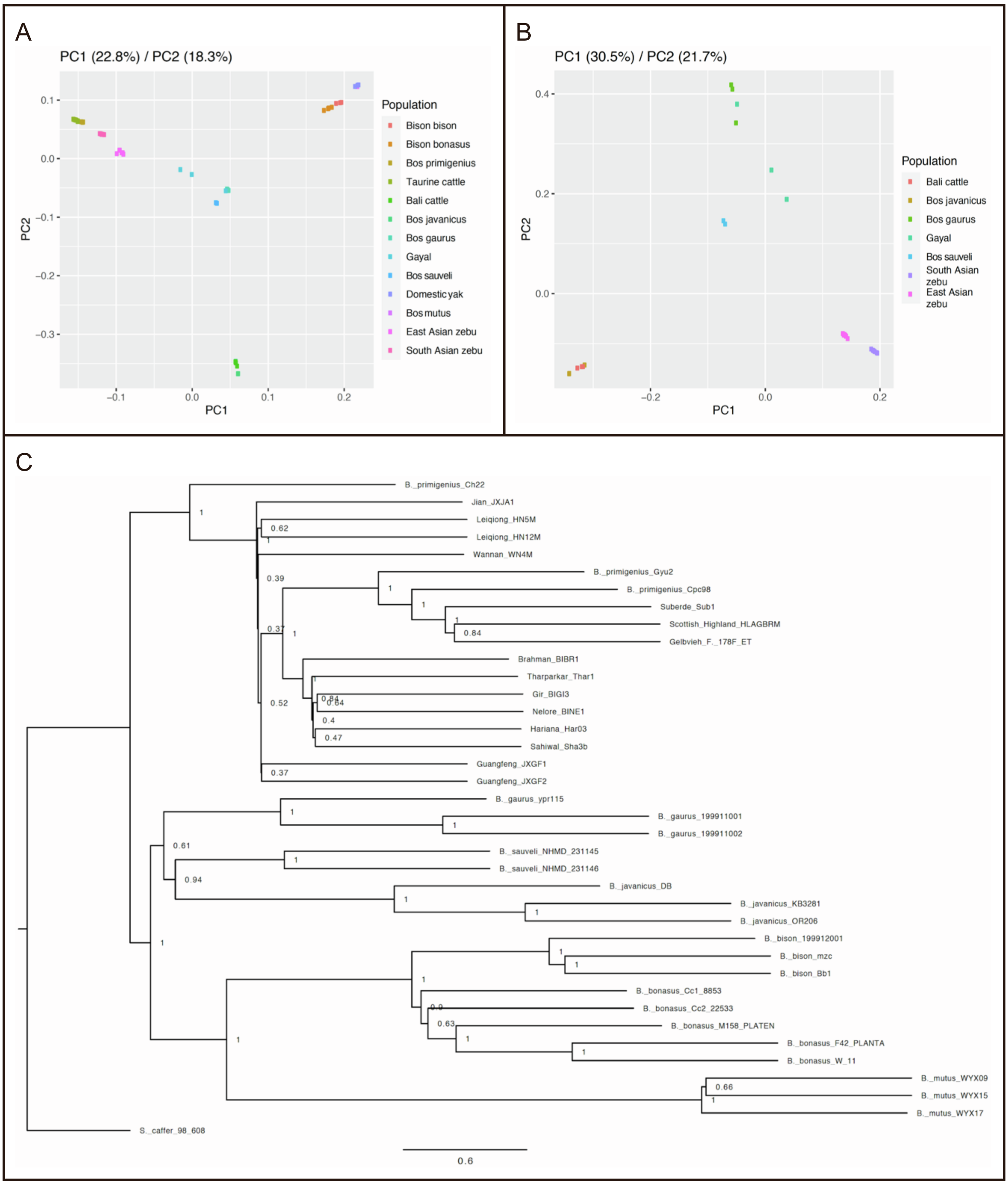

Figure S3. PCAs and uncollapsed Astral phylogeny.

A PCA of full nuclear genomes. **B** PCA of full nuclear genomes excluding Yak. PCA analysis based on genotype likelihoods estimated in PCAangsd, including all genomes (Table 1 and Table S1). **C** Uncollapsed Astral phylogeny of full nuclear genomes. Number basal to each node represents the posterior probability that supports the topology shown here. Names of specimens given at the end of each branch. Branch lengths indicate coalescent times, but do not correspond to the actual time between clades. Related to ”Main figure 2”.

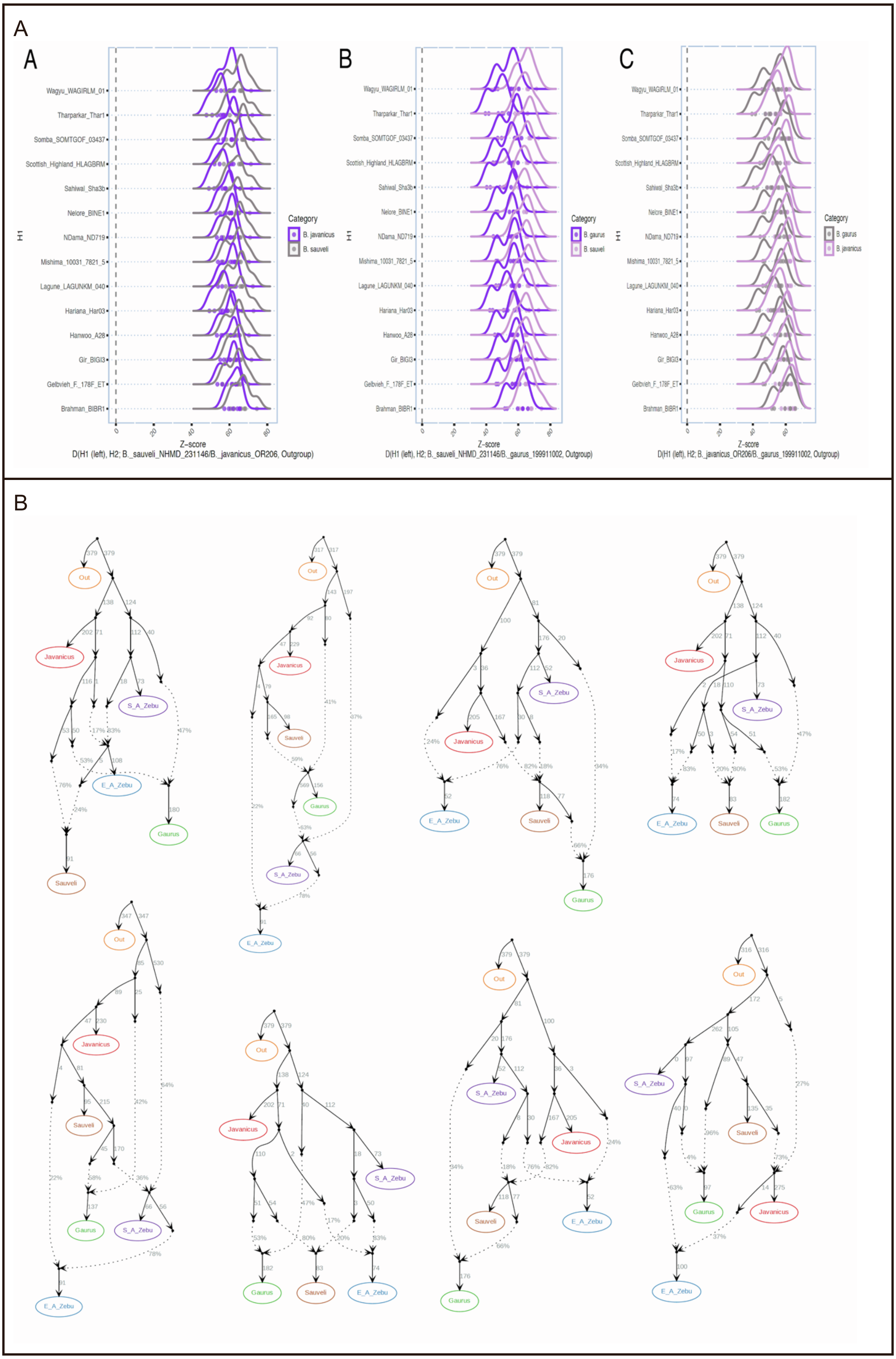
A
A
B
C
Wagyu_WAGIRLM_01
Tharparkar_Thar1
Somba_SOMTGOF_03437
Scottish_Highland_HLAGBRM
Sahiwal_Sha3b
Nelore_BINE1
NDama_ND719
Mishima_10031_7821_5
Lagune_LAGUNKM_040
Hariana_Har03
Hanwoo_A28
Gir_BIGI3
Gelbvieh_F_178F_ET
Brahman_BIBR1
H1
Category
B. javanicus
B. sauveli
B. gaurus
Z-score
D(H1 (left), H2; B._sauveli_NHMD_231146/B._javanicus_OR206, Outgroup)
D(H1 (left), H2; B._sauveli_NHMD_231146/B._gaurus_199911002, Outgroup)
D(H1 (left), H2; B._javanicus_OR206/B._gaurus_199911002, Outgroup)
B
Out
Javanicus
S_A_Zebu
E_A_Zebu
Gaurus
Sauveli

Figure S4. D-statistics and additional admixture graphs.
A Asian Bos allele sharing with East Asian zebu. D-statistics tests of gene flow from **AA.** *B. sauveli* contra *B. javanicus*, **AB.** *B. sauveli* contra *B. gaurus*, **AC.** *B. gaurus* contra *B. javanicus*, into East Asian zebu compared to South Asian zebu and taurine cattle. H2 in each test are Guangfeng_JXGF1, Guangfeng_JXGF2, Jian_JXJA1, Leiqiong_HN5M, Leiqiong_HN12M, Wannan_WN4M. Points represent the Z scores obtained from each test. Colors indicate H3 reference, detailed in sub-figure. All tests are significant with a score above a threshold $|Z| \geq 3.3$. B. sauveli yields the highest score in all tests, greatly indicating that the exotic ancestry in East Asian zebu is well explained as wild Asian *Bos*.
B Complementary admixture graphs. A selection of admixture graph from qpbrute run Subset_1 (Table S2). All models fit left no outliers ($|Z|>3$). The selection of figures is a qual fit of the data as the main figures 3E-F. Solid lines indicate genetic drift, dotted lines indicate gene flow. Related to ”Main figure 3”.
